# Effect of Scapular Function Training on Chronic Pain in the Neck/Shoulder Region: A Randomized Controlled Trial

**DOI:** 10.1007/s10926-013-9441-1

**Published:** 2013-07-06

**Authors:** Christoffer H. Andersen, Lars L. Andersen, Mette K. Zebis, Gisela Sjøgaard

**Affiliations:** 1 National Research Centre for the Working Environment, Lersø Parkalle 105, 2100 Copenhagen Ø, Denmark; 2 Institute of Sports Science and Clinical Biomechanics, University of Southern Denmark, 5230 Odense M, Denmark

**Keywords:** Rehabilitation, Scapula, Trapezius, Serratus anterior, Workplace, Intervention

## Abstract

*Purpose* Neck and shoulder complaints are common among employees in occupations characterized by intensive computer use. Treatment has varied from passive rest to active treatments and active treatments have often been divided into either training of the painful area or the surrounding musculature avoiding direct training of the painful area. Our study investigates the effect of the latter approach. The purpose of this study was in a randomised controlled trial to investigate if intensive scapular function training (SFT)—in terms of training of the lower trapezius and the serratus anterior muscle while minimizing direct training of the upper trapezius—is effective in reducing pain in adults with chronic non-specific pain in the neck/shoulder region. *Methods* 47 office workers with chronic non-specific pain in the neck/shoulder region were randomized to 10 weeks 3 × 20 min SFT with training supervision or to a control group. At baseline and at follow-up the participants were tested for maximum isometric shoulder strength by a blinded tester. Further, once a week participants reported pain intensity of the neck/shoulder during the previous week. *Results* In intention-to-treat analysis neck- and shoulder pain decreased 2.0 (95 % CI 0.35; 3.64) in SFT compared with control group (*p* < 0.05). Pressure Pain Threshold (PPT) increased 129 kPa in the lower trapezius in SFT compared with the control group (*p* < 0.01). Shoulder elevation strength increased 7.7 kg in SFT compared with the control group (*p* < 0.01) with no change in shoulder protraction strength. *Conclusions* SFT reduces pain intensity and increases shoulder elevation strength in adults with chronic non-specific pain in the neck/shoulder region. The magnitude of improvement in pain intensity was clinically relevant.

## Introduction

In many western countries chronic neck pain is highly prevalent among the working population [1]. Neck and shoulder pain can be associated with restricted joint range of motion and loss of muscle strength, and is among the most common conditions treated by physical therapists. The pathomechanisms of neck and shoulder pain are only scarcely known but it is associated with both psychosocial and physical factors [2]. One of the contributing factors to neck pain is repetitive work (e.g., computer) [3]. In women with chronic neck pain, several pathophysiological conditions such as impaired oxygenation and elevated lactate levels is seen within the painful muscle indicating overuse [4, 5]. Scapular dyskinesis is observed in patients with trapezius myalgia [6] and patients with shoulder disorders show an altered muscle activation balance towards increased upper trapezius activation and reduced serratus anterior activation [7–9]. Research have suggested that shoulder abnormalities and scapular dyskinesis may be linked to global weakness of the scapulothoracic muscles; others attribute scapular dyskinesis to scapular muscular imbalance rather than absolute strength deficits [10]. However, the causal chain of action has not been established. From both the scientific literature and physiotherapeutic experience it is proposed that excess activation of the upper trapezius, combined with decreased control of the lower trapezius and the serratus anterior contributes to neck/shoulder pain [10, 11]. It may apply both ways as Schulte et al. [12] found that experimentally induced pain in the biceps muscle increases trapezius EMG activity during sustained isometric contractions of arm muscles. A study by Lin and co-workers on persons with general shoulder dysfunctions found reduced posterior tilt in the scapula during four sub maximal functional work tasks compared with pain free controls, and attributed this to lower serratus anterior muscle activity [13]. The study also showed increased activation in the upper trapezius during two out of the four work tasks. A recent study found lower EMG activity in all muscles but the trapezius in response to repeated cognitive stress [14]. Where other muscles showed lower EMG activity as the stressful task was repeated this did not happen in the trapezius. A study by Samani et al. [15] showed increased activity in the upper parts of trapezius due to experimental pain during computer work. Thus, pain during computer work may led to altered muscle activation patterns worsening the pain symptoms and entering a vicious cycle.

In addition to massage therapy [16], several training strategies have been examined, ranging from cardiovascular training only involving non-painful muscles [17], all-round physical exercise [18], kettlebell training [19], proprioceptive/muscle coordination training [20, 21] to intensive strength training for the entire shoulder girdle [17, 20, 22–25]. Thus, several training strategies can have an effect. However, the most effective training strategies involve intensive strength training [26] and training at least 1–2 times 20 min weekly [27]. Although high-intensive training involving the painful muscles can be effective, it is also shown to acutely increase neck pain [17] and may therefore be a barrier for individuals who already have severe neck and shoulder pain. In order to counteract compensation patterns and specifically target neck/shoulder dysfunctions through training rehabilitation detailed knowledge of exercise-specific activation balance of the scapular muscles is required. For patients with a compensatory pattern in the scapular muscles, selective activation of the weaker muscle parts with minimal activity in the hyperactive muscles is an important component in the reduction of the compensation. For these patients many physical therapists recommend neuromuscular training with selective activation of the weaker muscle parts with minimal activity in the hyperactive upper trapezius muscle [10, 28]. As it is possible to keep this selective activation even at high training intensities [29] intensive scapular function training may be an effective training tool for neck/shoulder pain. This alternative approach that has not been tested in randomized controlled trials.

Our randomized controlled trial investigates the effect of scapular function training—i.e., intensive training of the lower trapezius and the serratus anterior muscle while minimizing direct training of the upper trapezius—on pain intensity in adults with chronic pain in the neck/shoulder region.

We hypothesized that scapular function training will (1) reduce pain in the neck/shoulder region and (2) increase protraction strength.

## Methods

### Design Overview

This randomized controlled trial was performed in Roskilde, Denmark from September to December 2010. The participants were recruited from administrative departments of a large university. The local ethics committee approved the study protocol (H–C-2008-103), and all of the participants gave their written consent to participate. The trial is registered in the ClinicalTrials.gov, number NCT01205542 and the protocol published prior to the completion of intervention period [30].

### Setting and Participants

An announcement with a short introduction and invitation text, together with a link to an internet-based questionnaire was send to office workers from the administrative section of the university. When 100 workers had replied positive regarding participation we closed for further recruitment based on a priori power calculations and drop out estimates, and estimates of pain frequency in the neck/shoulder region. Out of the 100 responders 8 subsequently declined to participate in the study. Inclusion criteria were pain intensity in the neck/shoulder during the previous month of at least 3 on a 0–9 scale [31]. Exclusion criteria were (a) hypertension (Systolic BP > 160, diastolic BP > 100) or cardiovascular diseases (e.g., chest pain during physical exercise, heart failure, myocardial infarction and stroke), (b) symptomatic herniated disc or severe disorders of the cervical spine, (c) postoperative conditions in the neck and shoulder region, (d) history of severe trauma, and (e) pregnancy, (f) other serious disease. Further, the participants went through a clinical neck and shoulder investigation by a physical therapist [32] to exclude individuals with serious musculoskeletal disease. This lead to exclusion of one participant (generalized myalgia and radiating pain). The remaining sample consisted of 47 women and 10 men with a mean age (SD) of 44 (12) years, Body Mass Index (BMI) of 25 (4) kg m^−1^, 183 (136) days with pain in the neck/shoulder region within the last year and a baseline pain in the neck/shoulder region during the last month of 5.6 (1.7). All participant reported to work with a computer either ‘most of the working hours’ or ‘3/4 of the working hours’. Baseline demographics are presented in Table 1.

**Table 1:** Baseline demographics for the participant

	All	Control	Training	*p*
N	47	23	24	
Women	37	18	19	
Men	10	5	5	
Age	44 (12)	45 (11)	44 (13)	0.86
Height	171 (7)	171 (8)	171 (7)	0.84
Weight	72 (12)	72 (12)	72 (13)	0.99
BMI	25 (4)	25 (4)	24 (3)	0.85
Neck and shoulder pain last month (0–9)	5.6 (1.7)	5.4 (1.5)	5.7 (1.9)	0.64
Days with neck and shoulder pain within the last 12 months	212 (119)	211 (126)	213 (115)	0.96

### Randomization and Interventions

**Fig. 1:**
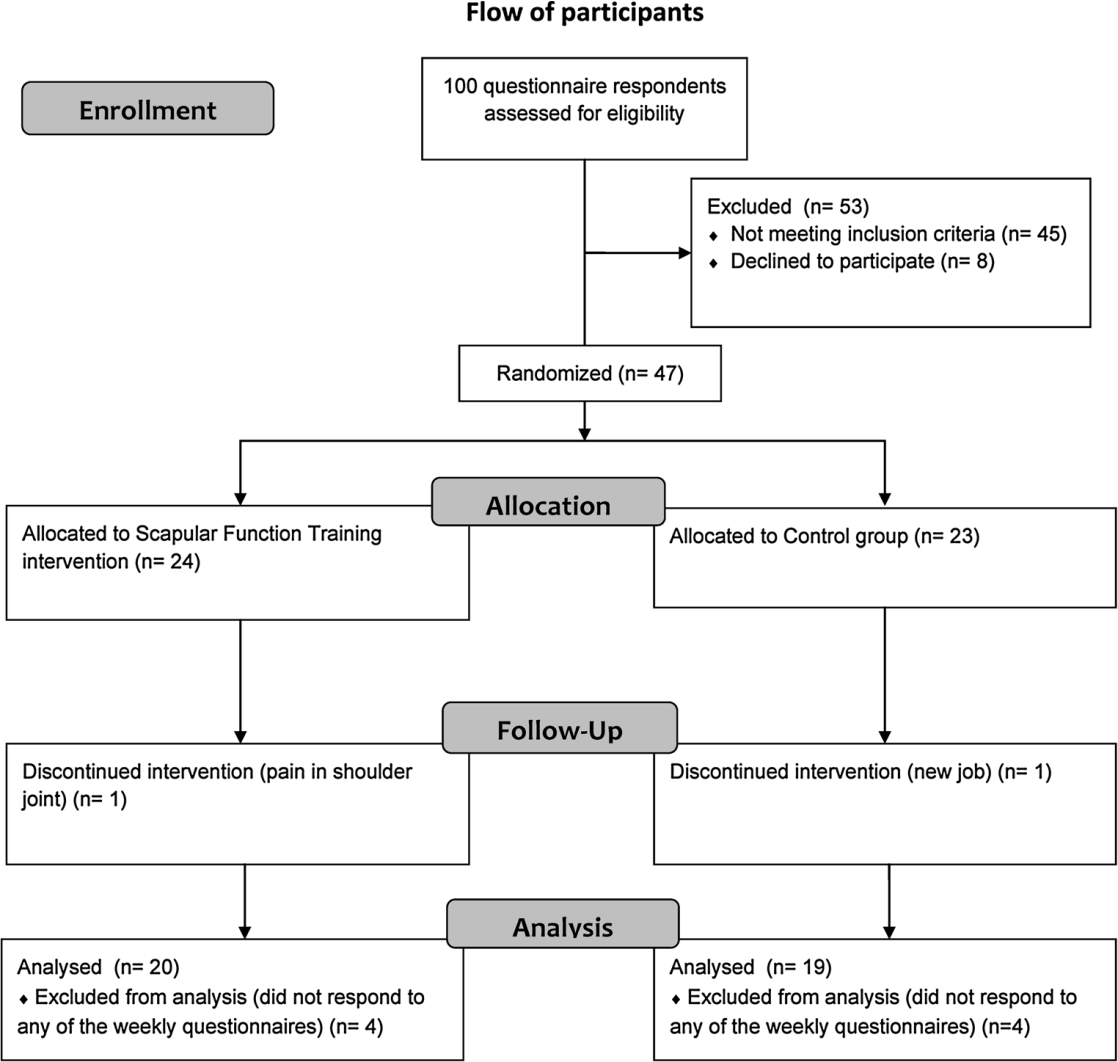
Flow of participants throughout the intervention

Using a computer generated random numbers table, the 47 participants were randomly allocated to SFT (n = 24) or Control (n = 23). Gender and age (44 and older or below 44 years) was used as stratification variables. There was no significant difference between the SFT and control group on any of the parameters at baseline [30]. Individuals with neck and shoulder pain were included as there is good correlation between both pain intensity and changes in pain intensity between the two [33] (Fig. 1).

**Fig. 2:**
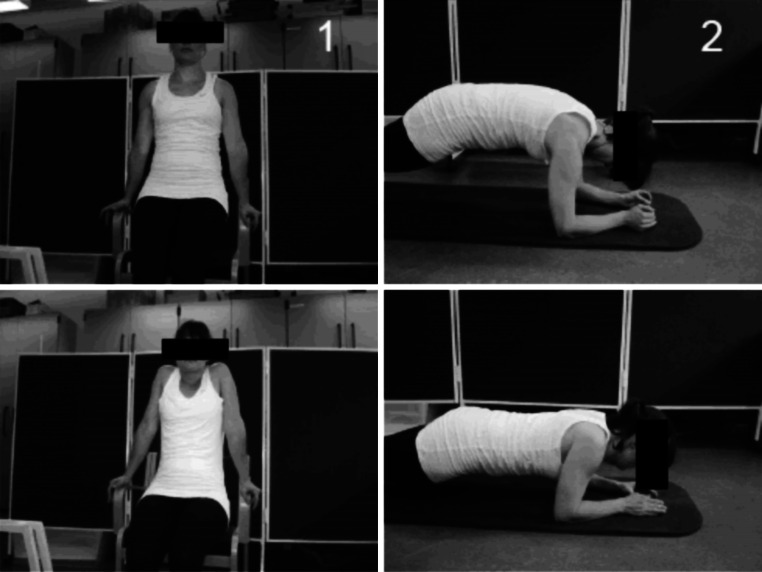
The two exercises used in the intervention (*1*) press-up and (*2*) push-up plus

The SFT group was allocated to 3 × 20 min training per week for 10 weeks during working-hours. An experienced instructor assisted in all of the training sessions. The control group was not offered any physical training but was encouraged to stay active as usual. The training-group performed scapular function training with exercises which have been shown to activate the serratus anterior and lower trapezius muscles to a high extent, but with only a low level of activation of the upper trapezius. The two exercises used were selected on the basis of previous study [29] and are pictured in Fig. 2. If needed, extra resistance was added by placing elastic bands of varying thickness across the back (push-up plus) or over the shoulders (press-up). During the 10-week intervention training load and volume were varied from approximately 20 Repetition Maximum (RM) in the first week to 10 RM in the last weeks according to the principle of periodization and progressive overload [34]. In the first week participants performed 3 sets of each exercise and worked up to a maximum of 5 sets of 10 repetitions in the last weeks of the intervention. Exercises were alternated in a ‘superset’ fashion. A superset is when you perform a set of one exercise then immediately, with only little rest, perform a set of a different exercise.

Each training session started with a short warm-up by slowly moving the neck, upper back, shoulder blades and shoulder joint through pain-free range of motion.

If a participant experienced incidence of joint pain or the like during a specific exercise, we asked them to adjust the exercise as follows: First, slightly alter the path or range of movement during the exercise. Then, the participant reduced the training load of the exercise. If this did not help, the participant reduced the number of sets of the given exercise in the session.

### Outcomes and Follow-up

In this single-blinded study, the participants from both groups replied to questionnaires [30] and went through testing of muscle strength as described in the following. The primary outcome is change in pain of the neck/shoulders at 10 weeks. Secondary outcomes are maximal muscle strength and PPT [30].

#### Self-Rated Pain Intensity

A weekly email questionnaire inquired about the intensity of pain in the neck and shoulder area [35] was rated subjectively on a scale ranging from 0 to 9 in, where 0 indicated “no pain at all” and 9 indicated “worst possible pain” [31, 36].

#### Pressure Pain Threshold

PPT was measured in the following sites; (1) the muscle belly of the upper trapezius identified by palpation around the midway point between C7 and acromion -as this muscle is often a site of pain in neck/shoulder cases; (2) the muscle belly of the lower trapezius identified by palpation 2/3 down between angulus superior and the spinal attachment—as this muscle was directly trained; (3) middle part of the sternum –non-muscle reference; and (4) the muscle belly of the tibialis anterior—as a non-related muscle reference. Single measurements were taken at each location and rotating between locations until three measurements were recorded for each site [37]. PPT was measured before and after the intervention using an electronic pressure algometer (Algometer Type 2; Somedic, Hörby, Sweden) [38]. The diameter of the contact area was 10 mm, and the pressure was applied perpendicular to the skin at a speed of 30 kPa s^−1^. The participants marked the PPT by pressing a button when the sensation of “pressure” changed to “pain.” There was at least 1 min between the three measurements on each site, and the mean value was calculated.

*Maximal muscle strength* was assessed as an objective functionality measure by a maximum isometric supine shoulder protraction test and a seated shoulder elevation test against a pair of strain gauge dynamometers at baseline and at the end of the intervention (10 weeks) [39]. Maximal muscle strength has previously been shown to discriminate well between adults with and without neck and shoulder [40]. Six maximal tries with at least 1 min rest between were performed for both protraction and elevation. The highest value is reported as maximal strength for the given test.

*Adherence* At the supervised sessions participant adherence was registered. This was supplemented by the weekly email questionnaire where participants were asked how many SFT sessions (both supervised and un-supervised) they had performed during the last week.

### Statistical Analysis

Using the statistical software package IBM SPSS 19 (IBM, CA, USA), we performed analyses according to the intention-to-treat principle, i.e., including all randomized participants regardless of actual participation and missing replies, and imputed missing values by last observation carried forward and backward. We performed repeated measures analysis of variance to model change in pain during the intervention period in the neck/shoulder. In a post hoc analysis we used linear regression analysis from all log entries to determine the change in pain over time for each individual [17]. The level of significance was set to *p* < 0.05. Baseline results are presented as mean (SD) and changes from baseline to follow-up as means [95 % confidence intervals (CI)] unless otherwise stated. We performed test–retest reliability for the control group before and after the intervention using intraclass correlation (ICC) for the MVCs combined, PPTs combined and each test separately.

Power analyses performed prior to the study showed that—to reject the null-hypothesis of equality—we should include 20 participants per group (allowing for a 20 % loss to follow-up) for 80 % power and *p* = 0.05 to detect a clinically significant change in pain of 1.5 [31, 36] on a 0–9 scale between groups based on the pain ratings from the weekly questionnaire.

## Results

### Self-Rated Pain Intensity

**Fig. 3:**
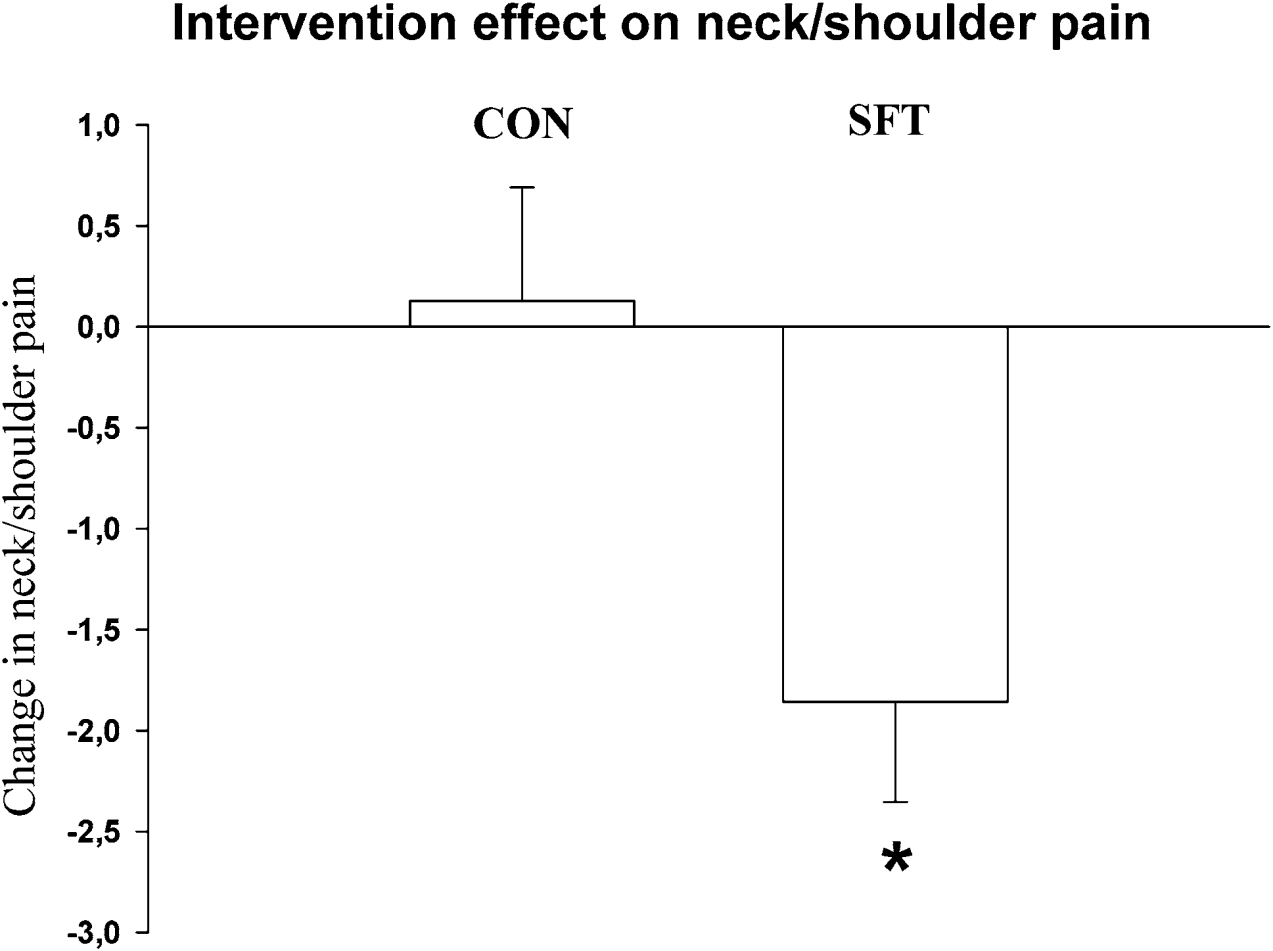
Difference in neck- and shoulder pain from baseline to 10 weeks follow-up. Values are means (SE). *between-group difference *p* < 0.05

At baseline the mean pain in the neck/shoulder region last month in the two groups were 5.4 (1.5) for control group and 5.7 (1.9) for SFT. The intention to treat analysis showed a group by time effect for the change from baseline to follow-up in pain in the neck/shoulder region between SFT and control group (*p* < 0.01). The post hoc test using linear regression for each individual showed a between-group difference 2.0 (95 % CI 0.4–3.6) over the 10 weeks as shown in Fig. 3.

### Pressure Pain Threshold

The analysis on pressure pain threshold in the lower trapezius showed a significant difference in change from baseline to follow-up between the two groups, where SFT had an increase of 129 kPa (95 % CI 31–227 kPa) (*p* < 0.01). There was no difference in the other regions (upper trapezius (95 % CI −53 to 158 kPa), sternum (95 % CI −14 to 158 kPa), and tibialis anterior (95 % CI −87 to 170 kPa). However, from baseline to follow-up the pressure pain threshold increased in all four sites in the SFT group where the control group did not change statistically significant (Table 1).

### Shoulder Elevation and Protraction Strength

From a baseline mean of 58.2 kg (15.3 kg), SFT increased shoulder elevation strength 7.7 kg (95 % CI 2.2; 13.3 kg) (*p* < 0.01) more than control group. The isometric protraction strength at baseline was 59.0 kg (20.2 kg). Although the mean difference in protraction strength at follow-up was 6.5 kg (95 % CI −3.5; 16.6 kg) higher in the SFT group compared to control group, this was not statistically significant.

### Test–Retest Reliability

The ICC on the control group measurements showed an acceptable correlation in both the MVC tests and the PPT measurements combined (Table 2). Only the PPT measurements for tibialis anterior had unacceptable reliability (Table 3).

**Table 2:** Within-group changes in pressure pain threshold from baseline to follow-up at each site

	Control group					SFT				
	Baseline (kPa)	SD	Follow-up (kPa)	SD	*p*	Baseline (kPa)	SD	Follow-up (kPa)	SD	*p*
Upper trapezius	303	(127)	378	(143)	NS	277	(155)	405	(186)	<0.05
Lower trapezius	383	(145)	399	(175)	NS	308	(162)	453*	(204)	<0.01
Tibialis anterior	381	(135)	464	(193)	NS	321	(93)	446	(165)	<0.05
Sternum	254	(154)	291	(124)	NS	225	(128)	323	(137)	<0.05

Values are means (SD)

* Between-group difference *p* < 0.01

**Table 3:** Test-retest reliability after 10 weeks for the control group measurements

	ICC coefficient	*p*
MVC combined	0.80	<0.001
Protraction	0.61	<0.01
Elevation	0.83	<0.001
PPT combined	0.52	<0.001
PPT upper trapezius	0.61	<0.01
PPT lower trapezius	0.83	<0.001
PPT sternum	0.75	<0.01
PPT tibialis anterior	0.36	NS

### Compliance/Adherence/Dropout

Mean adherence to the training was 2.1 (0.5) sessions per week. One of the participants in the SFT group dropped out after week four due to pain in the glenohumeral joint and one subject in the control group dropped out due to job change. However, these two participants are still included in the ITT analysis.

## Discussion

The present study shows that SFT—i.e., strength training of the lower trapezius and serratus anterior while minimizing activity of the upper trapezius—has a clinically relevant effect on chronic pain in the neck/shoulder region in adults.

From baseline to 10-week follow-up we found a between-group difference in pain intensity of 2 on a 10-point scale. Other studies using high-intensity strength training with several different neck/shoulder exercises targeting the deltoids, upper trapezius, neck extensors etc. reported pain reductions corresponding to approximately 1–3 on a 10-point scale [17, 24, 25]. Change in pain in the neck and shoulder region is considered clinically relevant when a statistically significant reduction of between 1.5 and 2 on a 10-point scale occurs [31, 41]. Our results show that SFT can be added to the clinically relevant treatment strategies for pain in the neck/shoulder region. This broadens the treatment options for these types of patients, e.g., some patients may not be able to directly train their upper trapezius due to severe pain but can still get clinically relevant reductions of pain from training the lower trapezius and serratus anterior.

In a workplace setting adherence to exercise programs is challenging [33], thus balancing the optimal physiological recommendations with practical solutions is necessary. A previous study from our group has shown that with traditional strength training for the neck and shoulder, combinations of 1 × 60, 3 × 20, and 9 × 7 min/week all provide benefits in terms of pain reduction with no statistical differences between groups [42]. For physical exercise to be feasible in a workplace setting, the exercise should be easy to implement in the daily routines as this has a marked effect on training adherence [43, 44]. The SFT exercises can easily be fitted into other time-wise setups and demands little training equipment besides the participants own body weight.

All previous interventions applying intensive muscle training have used exercises which—besides the targeted painful muscles—have shown to activate the majority of muscles in the shoulder girdle [45]. To our knowledge, this is the first intervention study on neck and shoulder pain using only exercises documented to selectively activate the serratus anterior and lower trapezius muscles at high intensities but with only a low activation of the upper trapezius [29]. This is also a practice that has been used within physical therapy targeting other disorders such as impingement syndrome, rotator cuff dysfunction, and instability [10, 28].

We measured PPT to get a more objective pain rating in contrast to the purely subjective VAS measure. It should be noted that PPT may only be considered “semi-objective”, because the participant still rates the pain threshold but is unaware of the actual figure when the threshold is met. The PPT recordings showed that pain sensitivity only decreased in the lower trapezius which had been trained in the SFT compared to the control group. It has previously been showed that mechanical hypoalgesia can be induced in painful muscles by exercising the muscle, regardless of exercise mode [37, 46]. Although only specific exercise seems to increase PPT of a painful muscle, earlier studies have showed that the PPT of pain-free reference muscles can increase in response to both specific and non-specific training, indicating a general effect of physical activity on pain perception [37, 47]. This is also supported by our study where PPT in the SFT group increased in all regions after the intervention. Thus, subjects with musculoskeletal pain may be able to modulate general pain perception in other body areas by training non-painful muscle groups [48].

It has been suggested that musculoskeletal disorders in the neck and shoulder region may be linked to weakness of the scapulothoracic muscles [17] or strength imbalance of these muscles [11]. The control group experienced an approximately 10 % increase in neck pain and decrease in shoulder elevation strength during the intervention period which can be contributed to progression of their painful condition, seasonal variation [33] and the inherent influence of pain inhibition on muscle strength [40].

As to the direct mechanisms behind the pain reduction we can only speculate, but increased muscular strength, altered motor patterns and increased body awareness may have led to relief of the overloaded and painful tissue.

## Limitations

What is considered clinically meaningful changes in pain for different body regions vary. Change in pain is considered clinically relevant for neck pain when a statistically significant reduction of at least 1.5 on a 10-point scale occurs [31, 36]. For shoulder pain, the change in pain is considered meaningful with a change of at least 2.2 on an 11 point scale [41]. However, a change of 2.0 on a 10 point scale as found in the present study would approximately translate to a change of 2.2 on an 11 point scale. Thus, even when we take the slight difference in resolution between the two scales into account the changes in pain is of a magnitude that falls within what is considered clinically significant for both shoulder and neck.

The study does not compare SFT to other active treatments and does not tell us if SFT is more effective against pain in the neck/shoulder region than other treatments.

One participant dropped out of the study due to discomfort in the shoulder joint experienced during training. No one else reported discomfort to the training instructor. However, there was not systematically collected information about discomfort experienced during training so it is not known how well tolerated the exercise program was.

## Conclusion

Our study confirms the first hypothesis that SFT reduces pain intensity in adults with chronic non-specific pain in the neck/shoulder region. However, we cannot confirm the second hypothesis that SFT would improve shoulder protraction strength. This may be due to low statistical power as the protraction test showed lower test–retest reliability than the shoulder elevation test.

In conclusion SFT reduces pain intensity and increases shoulder elevation strength in adults with chronic non-specific pain in the neck/shoulder region. The magnitude of improvement in pain intensity was clinically relevant.
